# Normalized vs factorized spectra: comparative spectrophotometric approaches for enhancing the resolution of zero-order absorption spectra of olanzapine and fluoxetine in mixtures

**DOI:** 10.1186/s13065-026-01722-3

**Published:** 2026-02-24

**Authors:** Khaled Hesham, Hend Z. Yamani, Shereen M. Tawakkol, Lobna A. Hussein, Nesma M. Fahmy

**Affiliations:** 1 Pharmaceutical Chemistry Department, Faculty of Pharmacy, Ahram Canadian University, 6th of october Giza, Egypt; 2 Pharmaceutical Analytical Chemistry Department, Faculty of Pharmacy, Ain Shams University, Cairo, 11566 Egypt

**Keywords:** Olanzapine, Fluoxetine, Constant multiplication method, Factorized zero order method, Normalized spectrum, Factorized spectrum

## Abstract

Simple, accurate, and precise UV spectrophotometric methods were developed and validated for the simultaneous determination of olanzapine (OLA) and fluoxetine (FLU) in bulk powders and combined pharmaceutical formulations without prior separation steps. The methods employed advanced mathematical manipulation techniques using both the Constant Multiplication method (CM) coupled with Spectrum Subtraction (SS) and the Factorized Zero-Order method (FZM), which was also coupled with SS. These approaches utilized normalized and factorized spectra, respectively, to resolve the overlapping spectral profiles of the two drugs. Unlike conventional methods reported in the literature, such as derivative spectrophotometry, our approach enabled the extraction of each component in its original zero-order form, exhibiting spectral features identical to those of pure standards, which enhances accuracy and precision. A direct comparative evaluation of CM–SS and FZM–SS is presented, highlighting their respective analytical merits. The proposed methods demonstrated linearity over concentration ranges of 1.5–14 µg/mL and 3.5–35 µg/mL for OLA and FLU, respectively, with limits of detection of 0.15 µg/mL (OLA) and 0.38 µg/mL (FLU), and limits of quantification of 0.47 µg/mL (OLA) and 1.15 µg/mL (FLU), confirming the high sensitivity of the methods. They were successfully applied for the simultaneous determination of OLA and FLU in synthetic mixtures and in their combined dosage form with excellent accuracy and precision. Furthermore, the environmental and practical merits of the methods were evaluated through the principles of Green Analytical Chemistry (GAC) and White Analytical Chemistry (WAC). The greenness of the proposed spectrophotometric method was evaluated by the Analytical Greenness Metric (AGREE). The overall method performance, including validation efficiency, ecological impact, and practicality, was systematically evaluated using the RGB-based WAC approach.

**Supplementary Information:**

The online version contains supplementary material available at 10.1186/s13065-026-01722-3.

## Introduction

Olanzapine (OLA) is an atypical antipsychotic medication widely used for the treatment of schizophrenia and bipolar disorder. Its therapeutic efficacy results from its antagonistic action on central dopaminergic and serotonergic receptors, leading to the modulation of neurotransmitter pathways involved in mood and cognition [1, 2]. OLA is frequently prescribed in combination with fluoxetine (FLU), a selective serotonin reuptake inhibitor (SSRI). The combination is indicated for managing bipolar depression and treatment-resistant major depressive disorder [3, 4]. Both drugs work by increasing the activity of serotonin, norepinephrine, and dopamine in the brain [5]. The synergistic effect of the two drugs helps stabilize mood (OLA) and reduce depressive symptoms (FLU). Therefore, this combination is an important treatment option in psychiatric care, necessitating the development of robust and reliable analytical methods for its simultaneous determination.

**Fig. 1:**
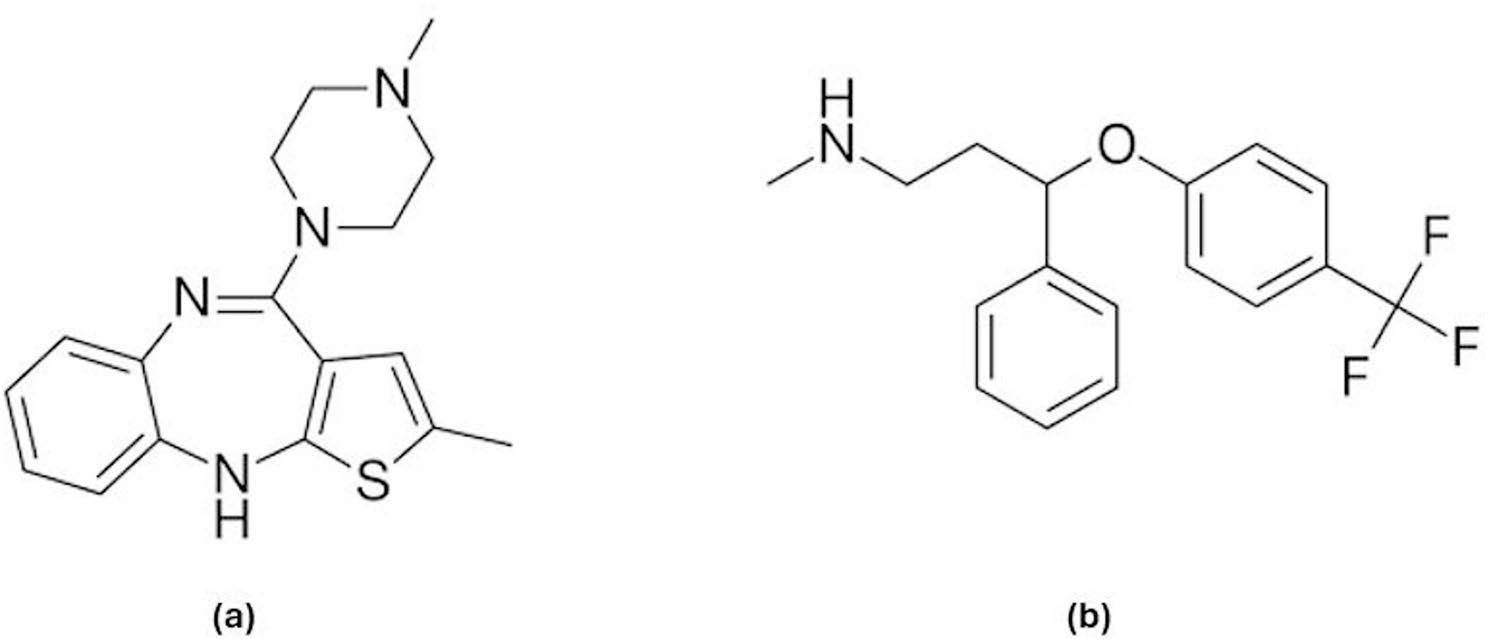
The chemical structure of (**a**) OLA and (**b**) FLU

Chemically, OLA is identified as 2-methyl-4-(4-methylpiperazin-1-yl)-10 H-thieno[2,3-b][1,5]benzodiazepine, whereas FLU is recognized as N-methyl-3-phenyl-3-[4-(trifluoromethyl)phenoxy] propan-1-amine [6, 7] (Fig. 1). OLA is a yellow crystalline solid that is practically insoluble in water [6] but practically soluble in methanol, while FLU is a white to off-white crystalline solid that is also insoluble in water but soluble in organic solvents such as methanol [8].

Several analytical methods have been reported for the simultaneous determination of olanzapine and fluoxetine in pharmaceutical formulations. These methods include chromatographic techniques such as HPLC with UV or mass spectrometric detection [9–16], TLC [17, 18], and various spectrophotometric approaches [17, 19–23]. Although chromatographic methods provide high sensitivity and selectivity, they often require expensive instrumentation, time-consuming sample preparation, and large volumes of organic solvents, which can limit their routine application in settings with limited resources and contradict principles of green analytical chemistry [24–28]. On the other hand, the reported spectrophotometric methods, such as first-derivative spectrophotometry [17, 22], derivative ratio [17], absorbance correction, absorbance ratio, simultaneous equation (Vierodt’s) method [21], ratio subtraction, and dual-wavelength techniques [23], have inherent drawbacks. Derivative-based approaches [17, 22] can amplify noise and distort spectral features, which can compromise precision and accuracy, particularly at low concentrations [29–33], while subtraction and correction methods may accumulate errors during multi-step processing. The simultaneous equation and absorbance ratio techniques [21, 34] are generally unreliable for complex mixtures. The simultaneous equation method requires distinct λmax values for each component with minimal spectral overlap; a condition rarely met in strongly overlapping mixtures such as olanzapine and fluoxetine. The method is highly sensitive to spectral interference, baseline noise, and slight shifts in wavelength, all of which compromise accuracy and selectivity. The absorbance ratio methods rely on a single isosbestic point, making them highly sensitive to minor wavelength or solvent changes, directly affecting method robustness [35]. Additionally, techniques like ratio subtraction, dual-wavelength [23], and absorptivity factor methods require strict spectral conditions, such as constant absorbance regions or equal absorptivity values, that are not always achievable in real mixtures [36]. These limitations reduce their applicability, making them less reliable for complex or severely overlapping spectra, which highlights the need for more reliable alternatives.

The proposed method comprehensively addresses the shortcomings of existing techniques. It is fully validated according to ICH Q2(R1) guidelines, demonstrating excellent accuracy and precision. Furthermore, statistical comparison against USP Official HPLC methods confirms the method is a valid, equivalent alternative. The proposed method is highly suitable for modern real-life applications due to its superior sensitivity, which is proven by the narrow linearity ranges for OLA and FLU. Combining high precision and speed, with minimal solvent consumption and waste, the technique provides clear advantages in sustainability and cost-per-sample for high-throughput quality control analysis. A comprehensive comparison between the proposed method and the reported methods has been illustrated in Table 5.

The objective of this study was to develop and validate simple, accurate, and eco-friendly UV spectrophotometric methods for the simultaneous determination of OLA and FLU in their pure forms and in combined dosage formulations. Specifically, we aimed to apply and directly compare the Constant Multiplication with Spectrum Subtraction (CM-SS) and Factorized Zero-Order with Spectrum Subtraction (FZM-SS) methods. These approaches have their own advantages; they enable the mathematical resolution of the severe spectral overlap by retaining spectra in the zero-order domain, thus reducing noise and preserving the original absorption characteristics. With high-level mathematical manipulation and spectrum subtraction, selectivity and resolution are improved without restrictive conditions, and even with mixtures of variable concentration ratios, accurate determination can be achieved. Furthermore, the simplicity and strength of these methods minimize the propagation of errors and extend their applicability, making them effective and powerful alternatives to traditional spectrophotometric methods. Additionally, the environmental and practical benefits of both proposed methods was assessed and quantified using the principles and metrics of Green Analytical Chemistry (GAC) and White Analytical Chemistry (WAC).

### Theoretical background

#### Constant multiplication coupled with spectrum subtraction (CM-SS)

This technique is particularly effective for resolving mixtures of two or more components, where one component exhibits a more extended absorption spectrum than the other. It combines mathematical normalization with spectral subtraction to separate overlapping spectra without derivative transformation.

- Generation of the Normalized Spectrum

The normalized spectrum of the extended component (Y) in the mixture is obtained by dividing each of its zero-order absorption spectra by the corresponding concentration, and then averaging the resulting spectra.

This concentration-independent, averaged profile—designated as Y′— represents the spectral characteristics of pure Y and serves as a digital divisor.

b. Application of CM-SS

The CM approach [37, 38] allows the extraction of the zero-order absorption spectrum of the extended component in the mixture.

- To analyze a binary mixture of components X and Y, where Y has the more extended spectrum, the zero-order spectrum of the mixture (X + Y) was divided by the normalized spectrum Y′.

The division yields a new spectrum according to the following relationship:

$$\left( {{\mathrm{X}}\,+\,{\mathrm{Y}}} \right)/{\mathrm{Y}}^\prime {\text{ }}={\text{ X}}/{\mathrm{Y}}^\prime {\text{ }}+{\text{ Y}}/{\mathrm{Y}}^\prime $$  

$$\left( {{\mathrm{X}}\,+\,{\mathrm{Y}}} \right)/{\mathrm{Y}}^\prime {\text{ }}={\text{ X}}/{\mathrm{Y}}^\prime {\text{ }}+{\text{ Constant}}$$  

Y/ Y′ is constant over the region where Y is more extended and appears as a straight line parallel to the wavelength axis.

The first component (Y) is obtained by multiplying Y′ divisor by the constant obtained from this plateau region, recovering the D^0^ spectrum of Y.

The concentration of Y is then calculated using the corresponding regression equation obtained by plotting the absorbance values of its D⁰ spectrum at its λ_max_ against the corresponding concentrations.

The second component (X) is resolved by using the spectrum subtraction (SS) approach [39]. This involves subtracting the obtained D⁰ spectrum of Y from the original spectrum of the mixture (X + Y), resulting in the isolated D^0^ spectrum of X as described by the following equation:

$$\left( {{\mathrm{X}}\,+\,{\mathrm{Y}}} \right) - {\mathrm{Y}}\,=\,{\mathrm{X}}$$

Then we can calculate the concentration of X from the corresponding regression equation that is obtained by plotting the absorbance values of the D^0^ spectra of X at its λ_max_ against the corresponding concentrations.

#### Factorized zero-order method (FZM) coupled with spectrum subtraction (SS)

This technique is particularly useful for resolving overlapping spectra in binary mixtures where a certain wavelength at which one component has no contribution can be identified.

- Generation of factorized spectrum in FZM

This factorized spectrum of component Y—designated as Y_f_— is obtained by dividing the zero-order absorption spectrum (D^0^) of pure Y by its recorded absorbance value at the chosen wavelength (λzero point), where X doesn’t show any contribution [40–45]. This can be described by the following equation:

$${{\mathrm{Y}}_{\mathrm{f}}}={\text{ Y}}/{{\mathrm{A}}_{\mathrm{Y}}}(\lambda {\mathrm{zero}})$$

Where Y_f_ represents the factorized spectrum of component Y, and A_Y_​(λzero​) is the absorbance of Y at the zero-contribution wavelength.

b. Application of FZM-SS.

For a mixture containing X and Y, the absorbance of the mixture at λzero is given by:

${{\mathrm{A}}_{{\mathrm{mix}}}}(\lambda {\mathrm{zero}})\,=\,{{\mathrm{A}}_{\mathrm{Y}}}(\lambda {\mathrm{zero}})$Since X doesn’t show any contribution at this wavelength, the D^0^ spectrum of (Y) in the mixture can be recovered by multiplying the absorbance at the λzero point in the mixture by the corresponding factorized spectrum of Y according to:

${{\mathrm{Y}}_{{\mathrm{recovered}}}}={{\mathrm{A}}_{{\mathrm{mix}}}}\left( {\lambda {\mathrm{zero}}} \right){\text{ }} \times {\text{ }}{{\mathrm{Y}}_{\mathrm{f}}}$The D^0^ spectrum of (X) in the mixture can be obtained then by subtracting the recovered Y spectrum from the original mixture spectrum using the SS method:

$\left( {{\mathrm{X}}\,+\,{\mathrm{Y}}} \right)\, - \,{{\mathrm{Y}}_{{\mathrm{recovered}}}}={\text{ X}}$Finally, the concentrations of X and Y are then calculated from the corresponding regression equations.

## Materials and methods

### Instrumentation

A double-beam UV/Visible spectrophotometer (Model V-760, Jasco, Japan) was used to perform spectrophotometric measurements. Spectra manager^®^ software (JASCO Corporation, Version 2) was used for the acquisition and processing of spectra. Absorption spectra of solutions were measured in 1.00 cm quartz cuvettes at room temperature in the 200–400 nm wavelength range.

### Chemicals and reagents

#### Pure samples

OLA and FLU were supplied by October Pharma. Their purities were found to be 100.72 ± 0.85% for OLA and 100.25 ± 0.55% for FLU, respectively, according to the official USP methods.

#### Pharmaceutical formulation

Psycholanz^®^ capsules, each claimed to contain 25 mg FLU and 6 mg OLA, hard gelatin capsule, were manufactured by Mash for Pharmaceutical Industries, and purchased from the Egyptian market.

#### Solvents

Analytical grade methanol and distilled water were used throughout the study. The final analytical solvent was a mixture of methanol: water (50:50, v/v).

### Preparation of standard stock solutions

A standard stock solution (100.00 µg/mL) was prepared by accurately weighing and transferring 10 mg of each drug into a 100 mL volumetric flask, then dissolved and diluted to the mark with methanol: water (50:50, v/v).

### Preparation of calibration standards

Aliquots equivalent to the concentrations of 1.50–14.00 µg/mL for OLA and 3.50–35.00 µg/mL for FLU were transferred from their respective stock solution (100.00 µg/mL) into two series of 10-mL volumetric flasks. The volume was completed by adding methanol: water (50:50, v/v) to the mark.

### Procedure

#### OLA and FLU spectra

Zero-order absorption spectra of 10.00 µg/mL of OLA and FLU were recorded by scanning over the range 200–400 nm against methanol: water (50:50, v/v) as a blank.

#### Selection of key wavelengths

Based on the D^0^ spectra, a λ_max_ of 226 nm was selected for both OLA and FLU. A wavelength of 282 nm was selected as a zero-contribution point where only OLA has a distinct absorbance, while FLU shows no contribution.

#### Linearity and calibration curves

The calibration standards of OLA (1.50–14.00 µg/mL) and FLU (3.50–35.00 µg/mL) were scanned against a blank consisting of 50:50 (v/v) methanol and water mixture over the wavelength range of 200–400 nm. All stock and working solutions were prepared in methanol/water and used within the same day, under which conditions solution instability is not expected to significantly affect absorbance accuracy.

##### Obtaining regression equations

Zero-order absorption spectra (D^0^) for OLA and FLU were recorded directly. Calibration curves were constructed by plotting the absorbance at λ_max_ = 226 nm versus the corresponding concentration. Regression equations for each drug were obtained to be used for quantification.

Both CM-SS and FZM-SS are spectral resolution techniques applied to the same validated UV spectrophotometric method; therefore, a single linearity study and set of regression equations were used for quantification in both approaches.

#### Application of the proposed spectrophotometric methods for the determination of OLA and FLU in laboratory-prepared mixtures

Laboratory-prepared mixtures were analyzed using both resolution approaches (CM-SS and FZM-SS), then the corresponding regression equations were used for quantification following spectral resolution.

Approach 1: Constant Multiplication with Spectrum Subtraction (CM-SS) Method:

A. Preparation of OLA normalized spectrum:

Each OLA zero-order absorption spectrum (D^0^) was divided by its corresponding concentration.

The resulting profiles were then averaged to obtain the normalized spectrum (Y′) of OLA as a divisor.

B. Analysis of laboratory-prepared mixtures using the CM-SS method:

Laboratory-prepared mixtures containing different ratios of OLA and FLU were analysed. The mixture’s zero-order (D^0^) spectrum was divided by the D^0^ normalized spectrum of 1 µg/mL OLA.

The division yields a new spectrum showing a constant region (280–300 nm) where OLA is more extended.

Recovery of OLA spectrum:

The constant obtained from the extended wavelength region (280–300 nm) was then multiplied by the D^0^ normalized of 1 µg/mL OLA spectrum to recover its D^0^ spectrum.

Recovery of FLU spectrum by Spectrum Subtraction (SS):

The zero-order (D^0^) spectrum of FLU was obtained by subtracting the recovered OLA spectrum from the corresponding mixture spectrum.

The concentrations of both OLA and FLU were determined by measuring the absorbance at their respective λ_max_ (226 nm) in each recovered spectrum and substituting into their corresponding regression equations.

Approach 2: Factorized Zero Order with Spectrum Subtraction (FZM-SS) Method:

A. Preparation of OLA factorized spectrum:

The factorized spectrum of OLA was generated by dividing the D^0^ spectrum of OLA by its absorbance value at 282 nm (the zero-contribution wavelength).

Recovery of OLA spectrum:

The absorbance of the mixture was measured at 282 nm. This absorbance was then multiplied by the factorized spectrum of OLA to recover its D⁰ spectrum in the mixture.

Recovery of FLU spectrum by Spectrum Subtraction (SS):

The recovered OLA spectrum was then subtracted from the mixture spectrum to get the zero-order (D^0^) spectrum of FLU.

The concentrations of both OLA and FLU were determined by measuring the absorbance at their respective λ_max_ (226 nm) in each recovered spectrum and substituting into their corresponding regression equations.

#### Application of the proposed spectrophotometric methods for the determination of OLA and FLU in a pharmaceutical formulation

The contents of ten Pscholanz^®^ hard gelatin capsules were weighed and finely powdered in a clean, dry mortar.

A portion of the powder equivalent to a single capsule was quantitatively transferred into a 100 mL volumetric flask, extracted and completed to the mark with a mixture of methanol: water (50:50, v/v).

The solution was sonicated for 10 min, then filtered to obtain a stock solution with claimed concentrations of 250 µg/mL and 60 µg/mL for FLU and OLA, respectively.

An aliquot of 1.0 mL from the filtrate was transferred into a 10 mL volumetric flask and completed to the final volume with methanol: water (50:50, v/v), yielding a solution with claimed concentrations of 25 µg/mL FLU and 6 µg/mL OLA, equivalent to the labeled content per capsule.

This solution was analyzed following the same procedure used for the assessment of synthetic mixtures.

## Results and discussion

### Theory

For both OLA and FLU, the zero-order absorption spectra show a λ_max_ at 226 nm. To resolve this severe overlap, we developed two approaches: the Constant Multiplication Method (CM) coupled with Spectrum Subtraction (SS), and the Factorized Zero Order Method (FZM), also coupled with SS. In contrast to methods that are derivative-based, these proposed methods here allowed the direct extraction of each component in its original zero-order form, with spectral profiles that perfectly match those of pure standards. This not only enhanced selectivity but also increased reliability in quantitative analysis by preserving the native spectral features without the distortions commonly introduced by mathematical manipulations.

It is acknowledged that, unlike the simultaneous equation or simple derivative methods, CM-SS and FZM-SS are not currently recognized as official methods in major pharmacopeias. Therefore, the proposed methods should be considered validated academic contributions and alternative approaches for the routine quality control of olanzapine and fluoxetine. Their value lies in providing a validated, high-performance, and economically superior alternative to complex separation techniques (like HPLC) for laboratories seeking rapid, green, and cost-effective analysis.

While both methods successfully extract the D^0^ spectrum, they rely on different spectral conditions, offering laboratories two validated choices that are preferable under different scenarios. CM-SS requires the presence of an extended spectral region where only one component contributes significantly, making it highly robust against minor baseline noise but dependent on a large spectral difference between the components. In contrast, FZM-SS requires a single, discrete wavelength where the interfering component has zero absorbance, making it simpler and faster to execute. By providing this direct comparative evaluation, the study establishes a more comprehensive and flexible analytical strategy, demonstrating that the high selectivity of Spectrum Subtraction can be reliably achieved via two distinct, powerful mathematical routes.

This study was conducted on Psycholanz^®^ capsules, a pharmaceutical formulation containing both OLA and FLU. The objective of this study is the filtration of each D° spectrum alone to measure each drug at its λ_max_. This was achieved by either the CM or FZM, both coupled with SS. These approaches enabled mathematical filtration of each component in the mixture in its zero-order form, producing spectral profiles typical of each individual pure component, thereby ensuring high accuracy and precision in the analysis of the synthetic mixtures and combined dosage form.

**Fig. 2:**
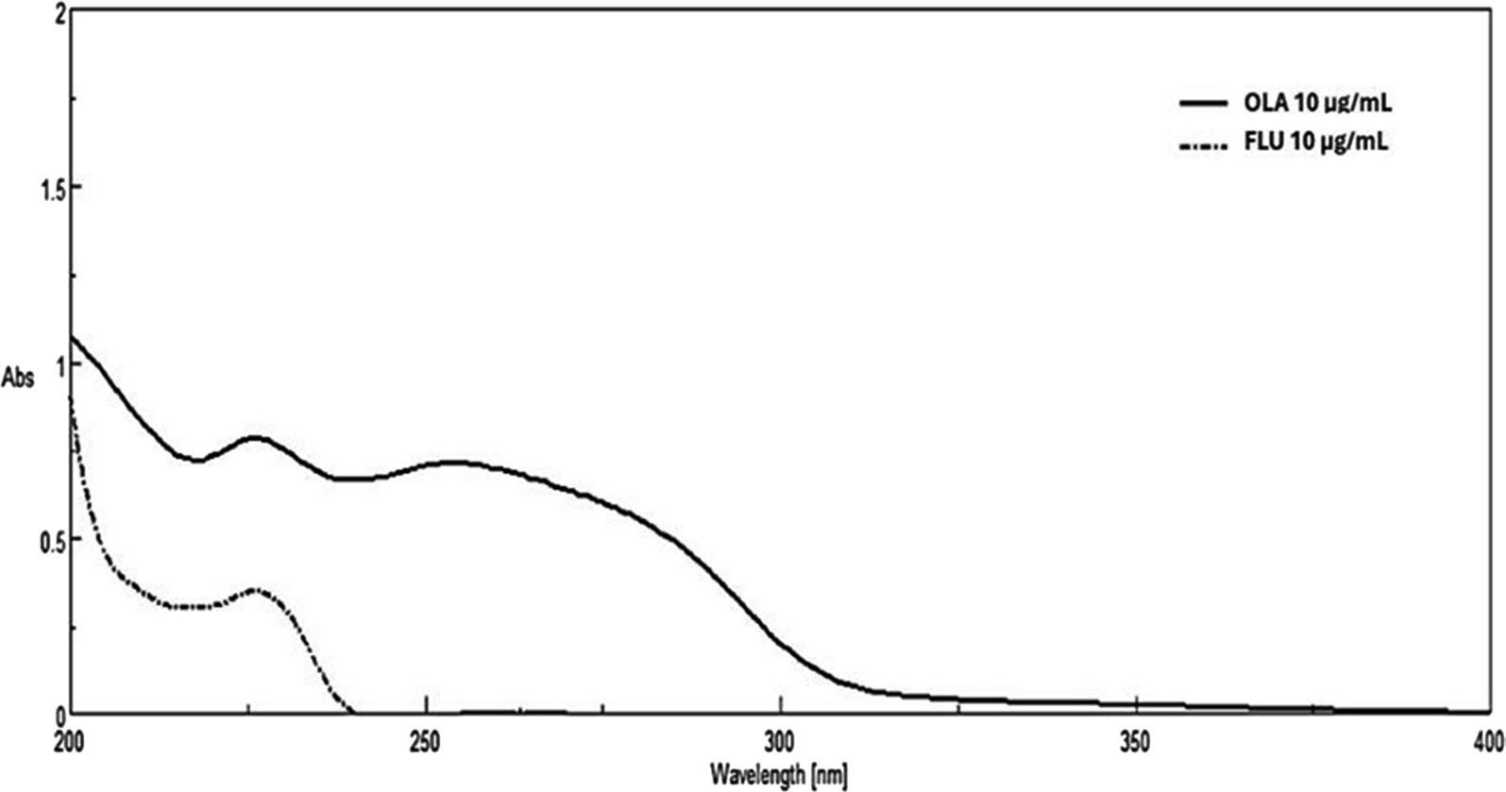
Zero order absorption spectra of OLA and FLU (10 μg/mL each) in methanol: water (50:50, v/v)

**Fig. 3:**
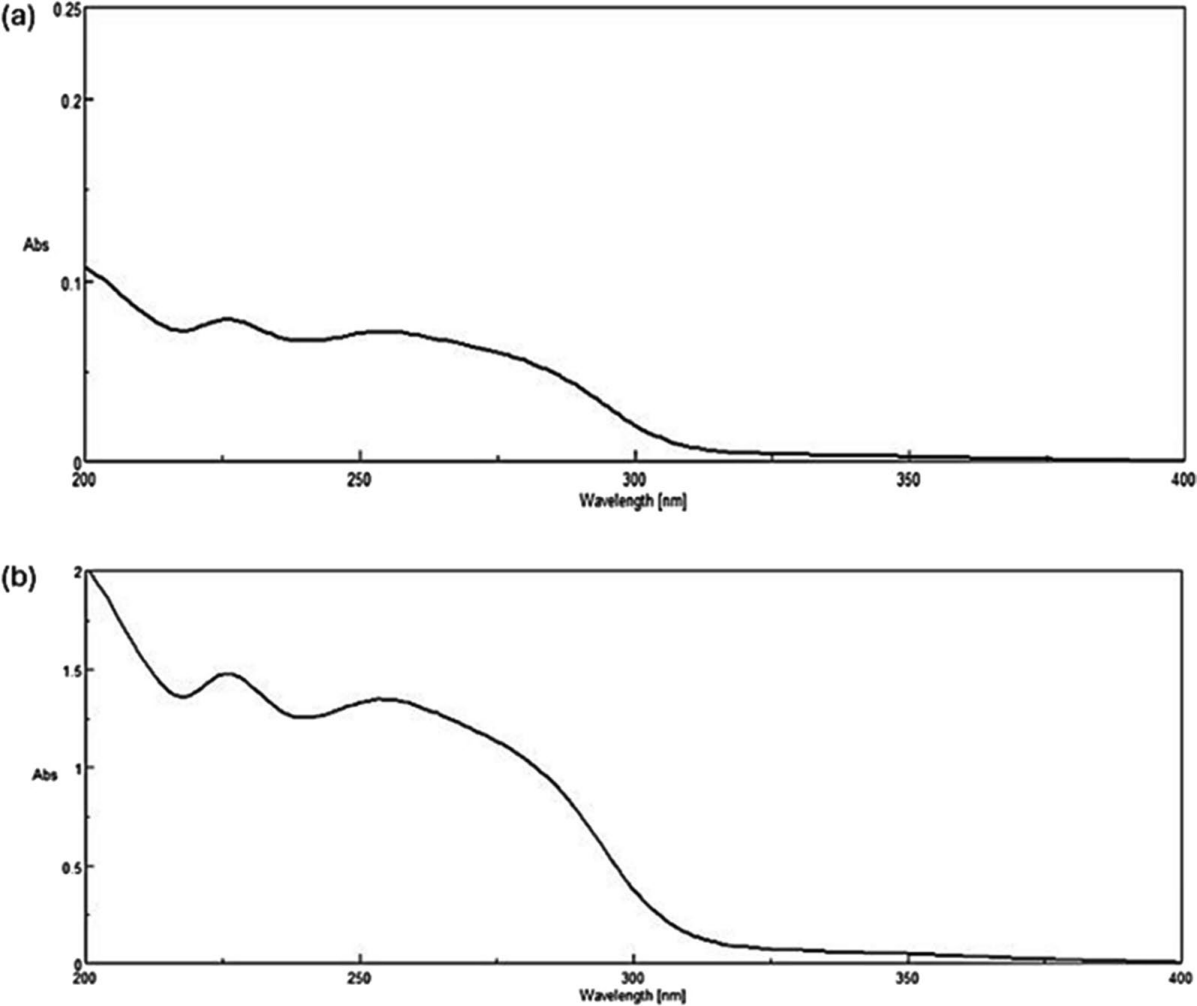
Normalized (**a**) and factorized (**b**) zero-order absorption spectra of olanzapine used for spectral resolution by CM-SS and FZM-SS approaches, respectively

**Fig. 4:**
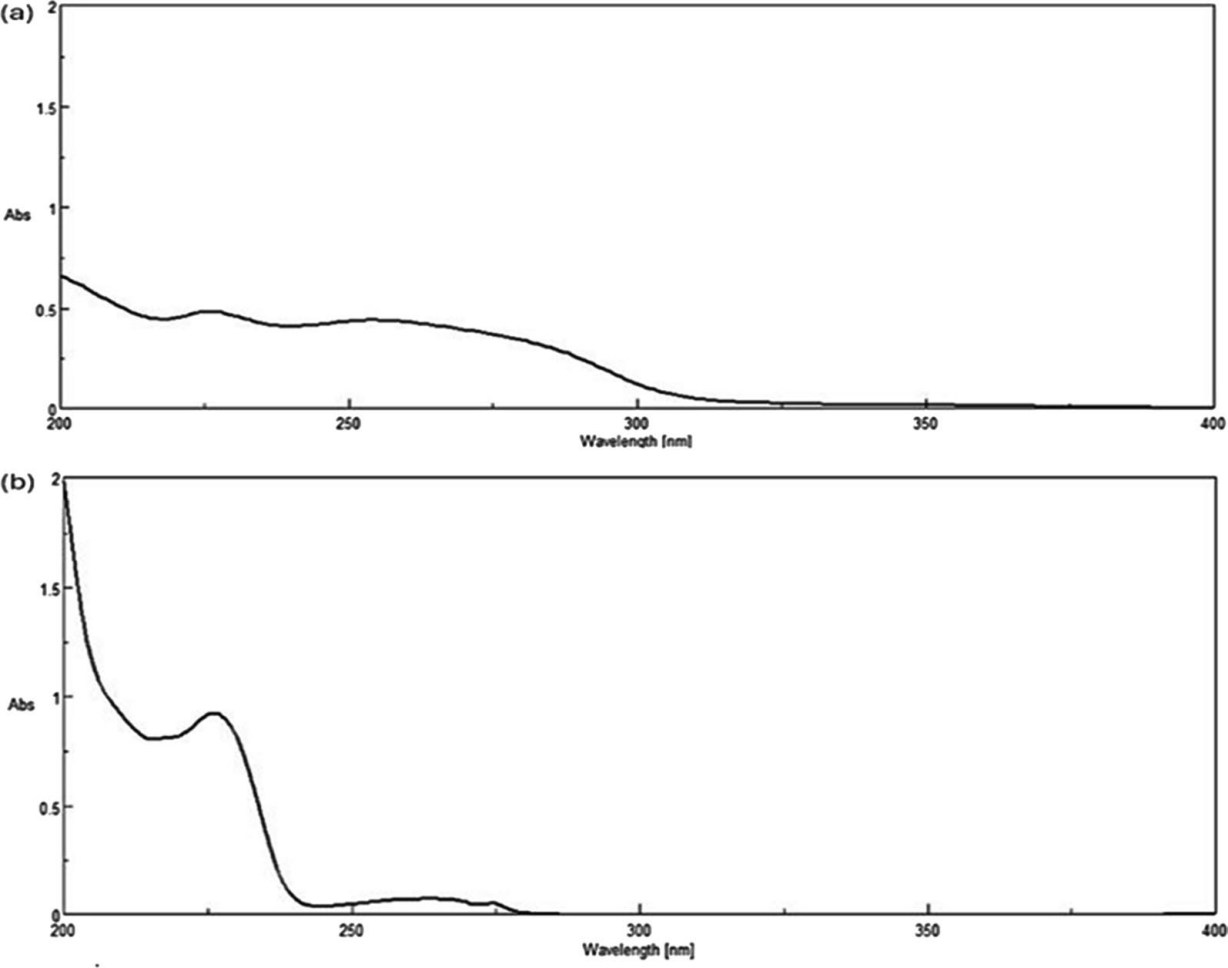
Zero-order absorption spectra of (**a**) olanzapine and (**b**) fluoxetine extracted from their laboratory-prepared mixture using CM-SS

**Scheme 1:**
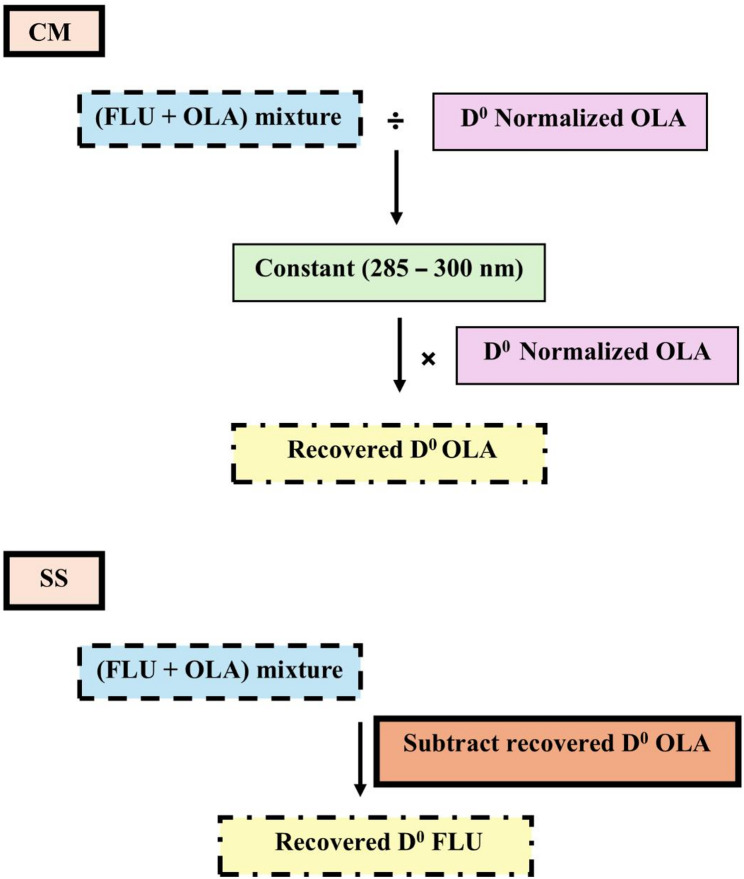
The manipulation steps of CM-SS method employed to extract the zero-order spectra of both drugs from the mixture

As shown in Fig. 2, the Dº absorption spectrum of OLA is more extended than FLU in the region 280–300 nm. Attempts to measure OLA directly at its λ_max_ (252 nm) didn’t succeed due to a minor contribution of FLU at this wavelength. However, by dividing the Dº absorption spectrum of the lab mixture by the Dº absorption spectrum of normalized 1 µg/mL OLA, a constant was obtained from the plateau region at 280–300 nm. This constant was then multiplied by the Dº absorption spectrum of normalized 1 µg/mL OLA to retrieve the Dº absorption spectrum of OLA present in the mixtures using the CM. The absorbance at λ_max_ 226 nm was then substituted in the regression equation to determine OLA concentration. Recovery of FLU by SS was obtained upon subtracting the recovered OLA spectrum from the binary mixture spectrum of both drugs (OLA + FLU). The concentration was then determined by substituting the absorbance at its λ_max_ (226 nm) in the corresponding regression equation (Scheme 1) as shown in Figs. 3 and 4.

**Fig. 5:**
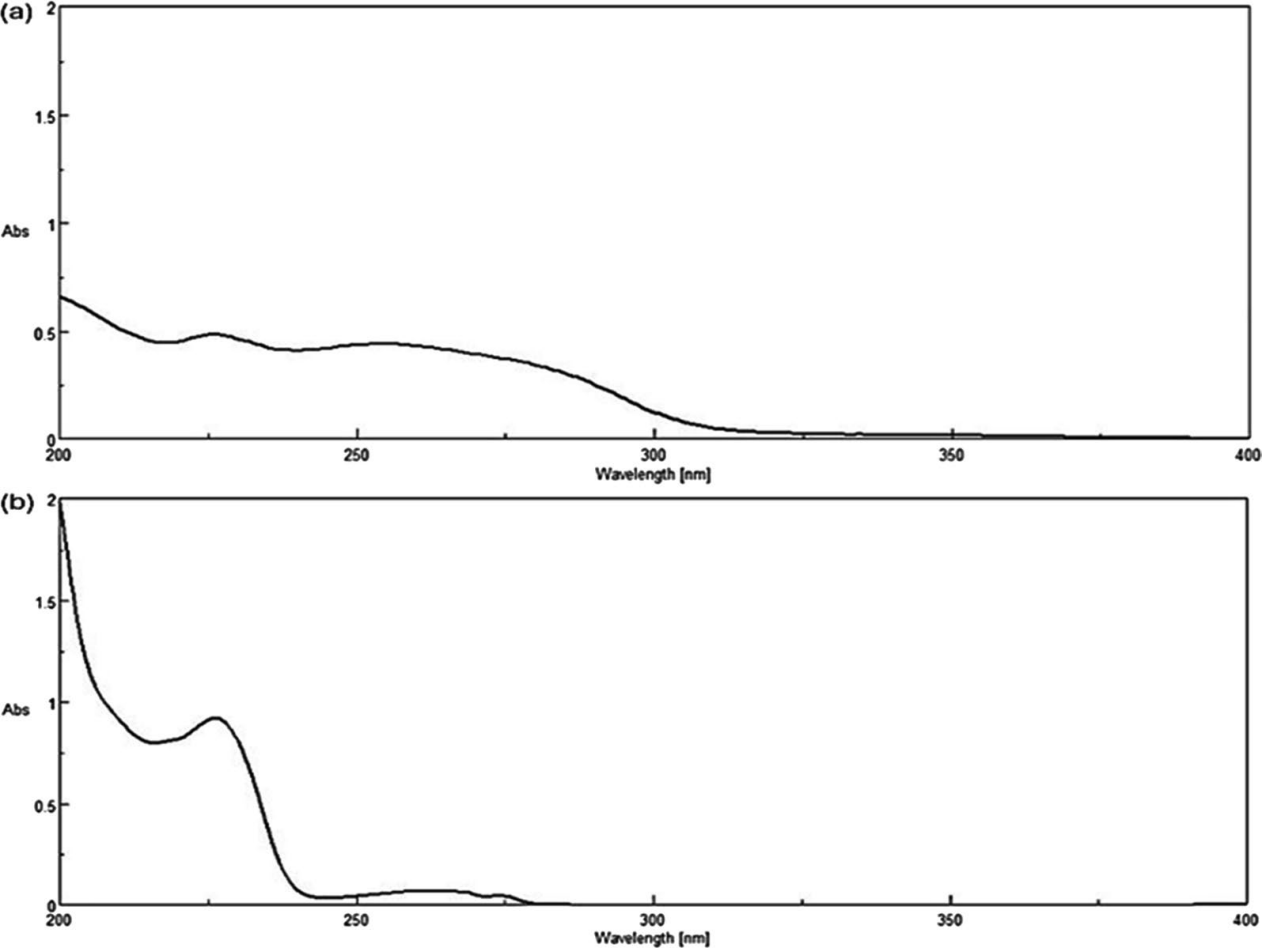
Zero-order absorption spectra of (**a**) olanzapine and (**b**) fluoxetine extracted from their laboratory-prepared mixture using FZM-SS

**Scheme 2:**
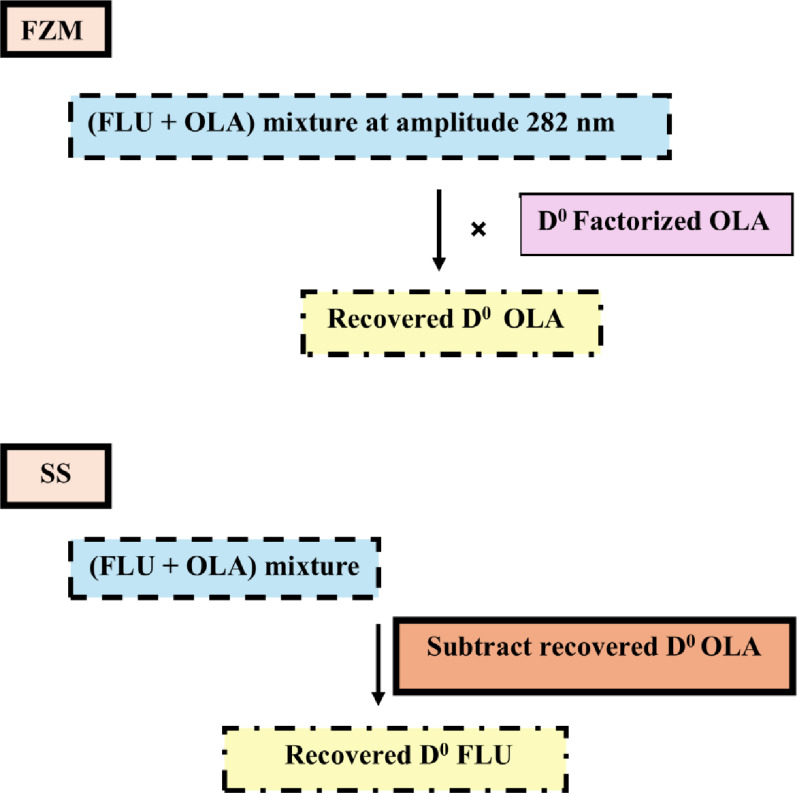
The manipulation steps of the FZM-SS method employed to extract the zero-order spectra of both drugs from the mixture

In the zero-order absorption spectrum of the drug combination, only OLA exhibits a distinct absorbance at 282 nm, as shown in Fig. 2. The FZM-SS method was applied, in which the absorbance of the binary mixture at 282 nm represents only the contribution of OLA, as FLU shows no contribution at this wavelength. This absorbance value was then multiplied by the factorized OLA spectrum. This allowed mathematical filtration of the zero-order spectrum (D^0^) of OLA from the mixture, which enabled its quantification at its λ_max_ (226 nm). We then subtracted the recovered D^0^ spectrum of OLA from the binary mixture (OLA + FLU), yielding the zero-order spectrum (D^0^) of FLU (Scheme 2) Figs. 3 and 5.

From a theoretical perspective, the normalized spectrum–based CM-SS method demonstrates superior robustness and tolerance to noise. This is because the ‘constant’ value is obtained by averaging the ratio spectrum over an extended plateau region (280–300 nm). This mathematical averaging across multiple data points inherently minimizes the effect of random instrumental noise or minor wavelength shifts, making it the preferable choice when baseline stability or spectrometer calibration accuracy is a concern. The main prerequisite, however, is the existence of a component with a significantly more extended spectrum than the other.

On the other hand, the FZM-SS method is distinguished by its simplicity and speed. Its calculation depends only on a single, specific zero-contribution wavelength (282 nm), making the initial setup faster and requiring minimal mathematical processing beyond the precise reading at this point. This makes FZM-SS highly suitable for high-throughput QC analysis where speed is required. However, this simplicity introduces a vulnerability; the method’s accuracy is dependent on the integrity of the signal at this single point, making it less tolerant to localized interference or significant spectral drift compared to the averaging benefit seen in CM-SS.

In summary, both CM-SS and FZM-SS enabling direct recovery of the zero-order spectra of OLA and FLU, thereby preserving the native spectral profiles and avoiding the distortions commonly associated with derivative processing. The CM-SS approach relies on the existence of an extended selective region (280–300 nm) where only OLA contributes significantly. CM-SS particularly advantageous under conditions where spectrometer calibration or baseline stability may be suboptimal, provided that a sufficient spectral difference between the components exists. In contrast, FZM-SS depends on a single zero-contribution wavelength (282 nm), where FLU shows negligible absorbance and only OLA absorbs, this reliance on a single wavelength makes it more susceptible to localized noise, spectral drift, or interference than CM-SS. Thus, the two methods are complementary: CM-SS is preferable when robustness is prioritized, whereas FZM-SS is favored when operational simplicity and analytical throughput are the main considerations.

### Methods validation

Validation of the methods was performed in accordance with International Conference on Harmonization (ICH) [46] guidelines as follows:

#### Linearity

Linearity was evaluated by constructing several calibration curves over three consecutive days. The calibration curves were prepared within concentration ranges that are appropriate for application to the dosage form. Each data point was measured in triplicates. The regression equation parameters and the concentration ranges for the proposed methods are summarized in Table 1.

**Table 1:** Assay parameters and method validation obtained by applying the proposed zero-order UV spectrophotometric method used for quantification after spectral resolution by either CM-SS or FZM-SS

Parameter	OLA	FLU
D^0^ (nm)	226	226
Range (µg/mL)	1.5–14	3.5–35
Regression equation	y = 0.0766x + 0.019	y = 0.0378x − 0.0316
Slope	0.0766	0.0378
Intercept	0.019	0.0316
Correlation coefficient (*r*)	0.9999	0.9999
Accuracy (Mean ± SD)	99.65 ± 0.48	100.27 ± 0.70
Intraday precision (%RSD^a)^	0.090	0.249
Interday precision (%RSD^b^)	0.639	0.756
Robustness (%RSD^c^)	0.197	0.432
LOD (µg/mL)	0.15	0.38
LOQ (µg/mL)	0.47	1.15
Molar absorptivity (ɛ) (L⋅mol^− 1^⋅cm^− 1^)	23,929.84	11,692.67
Sandell’s sensitivity (SS) (µg.cm⁻² per 0.001 A unit)	0.01305	0.02646

%RSD^a^ & %RSD^b^: Intraday and interday precision were evaluated at three concentration levels for each drug (OLA: 4, 6, 12 µg/mL; FLU: 4, 14, 30 µg/mL), with each measured in triplicate (*n* = 3)

%RSD^c^: Robustness of the zero-order UV spectrophotometric method, evaluated by introducing small deliberate wavelength variations (± 1 nm) around λmax = 226 nm for OLA and FLU

#### Limit of detection (LOD) and limit of quantification (LOQ)

The LOD and LOQ for the proposed methods were determined, and the values are shown in Table 1. They were calculated using the equations: LOD = 3.3 × (σ/S) and LOQ = 10 × (σ/S), where σ is the standard deviation of the residuals and S is the slope of the calibration curve. The low LOD and LOQ values confirm the high sensitivity of the proposed methods.

#### Accuracy

The results were found to be accurate through implementing the suggested methods for the assessment of blind samples of OLA and FLU within the linearity range. The mean percentage recoveries imply acceptable accuracy as listed in Table 1.

#### Precision and robustness

Intra-day and inter-day precision for the methods were evaluated by assessing three different concentrations per drug, repeated on the same day or on three consecutive days, within the linearity range. The results are summarized in Table 1.

In addition, the robustness of the zero-order UV spectrophotometric method was evaluated by introducing small deliberate variations in wavelength (± 1 nm) around the selected λmax for OLA and FLU. Since both CM-SS and FZM-SS resolution techniques are applied to the same zero-order UV spectrophotometric measurements, this assessment reflects the intrinsic robustness of the UV method itself. The low %RSD values obtained confirm that the zero-order UV spectrophotometric method is robust under minor wavelength variations, as shown in Table 1.

Furthermore, robustness of the resolution techniques was evaluated separately for the CM-SS and FZM-SS methods. CM-SS showed higher robustness than FZM-SS. For the CM-SS approach, robustness was assessed by varying the wavelength used to obtain the constant within the extended plateau region (285–300 nm, ± 1 nm), which demonstrated very good robustness. In contrast, for the FZM-SS method, robustness was evaluated by small variations in the selected zero-contribution wavelength (282 ± 0.1 nm), showing good robustness. The corresponding mean recovery values and standard deviations for both resolution techniques are summarized in Table 6.

#### Specificity

Specificity was ensured by analysis of laboratory-prepared mixtures of OLA and FLU at different ratios within the linearity range. The mean ± SD values obtained reflected accurate recoveries, which indicated the ability of the proposed method to selectively quantify each drug in the presence of the other. The results are summarized in Table 2.

**Table 2:** Determination of OLA and FLU in laboratory prepared mixtures using the proposed spectrophotometric methods

Concentration (µg/mL)	OLA		FLU	
	CM D^0^ 226 nm	FZM D^0^ 226 nm	CM D^0^ 226 nm	FZM D^0^ 226 nm
Lab mixture concentration (FLU: OLA)	Recovery % ± SD			
20:7	100.33 ± 0.22	100.96 ± 0.22	99.24 ± 0.20	98.80 ± 0.20
25:6*	99.85 ± 0.34	100.31 ± 0.35	100.49 ± 0.32	100.27 ± 0.32
35:3	99.86 ± 0.34	100.75 ± 0.98	99.99 ± 0.31	99.59 ± 0.31
10:10	99.87 ± 0.66	100.24 ± 0.67	99.86 ± 0.59	99.10 ± 0.59
15:5	99.46 ± 0.36	100.30 ± 0.37	99.69 ± 0.33	99.13 ± 0.33
Dosage form (Psycholanz^®^) 25:6	Recovery % ± SD			
	99.80 ± 0.33	100.26 ± 0.33	100.44 ± 0.31	100.22 ± 0.31

*Dosage form ratio

Values are presented as mean ± SD (*n* = 3)

### Statistical comparison

Statistical comparison was achieved by evaluating the proposed spectrophotometric methods versus the USP official methods [47] for the drugs under investigation, as shown in Table 3. No statistically significant differences were observed between the proposed and official methods; this was confirmed by both *F*- and *T*-tests. Also, one-way ANOVA was conducted (Table 4), and the results showed that there are no statistically significant differences between the proposed and official methods in the assessment of OLA and FLU (Tables 5 and 6).

**Table 3:** Statistical comparison between results obtained by the proposed validated zero-order UV spectrophotometric method and the USP official methods [47] for the determination of OLA and FLU in pure form

Statistical parameters	OLA		FLU	
	D^0^ 226 nm	USP Official method^a^	D^0^ 226 nm	USP Official method^b^
Mean	100.35	100.19	99.98	100.01
S.D.	1.45	0.76	0.61	0.55
N	6	6	6	6
Variance	2.1025	0.5776	0.3721	0.3025
F test (5.05) (df = 5, 5)	3.64	–	1.23	–
Student’s t-test (2.23) (df = 10)	0.24	–	0.10	–

^a^ HPLC conditions: Mobile phase: acetonitrile : buffer [prepared by dissolving 6.9 g of monobasic sodium phosphate in 1 L of water, adjusting the pH to 2.5 with phosphoric acid, and dissolving 12 g of sodium dodecyl sulfate in the resulting solution] (47:53, v/v); column: C18, 4.6 mm × 150 mm, 5 μm particle size; detection: UV at 260 nm

^b^ HPLC conditions: Mobile phase: tetrahydrofuran : methanol : buffer [triethylamine : water (1:98), adjusted to pH 6.0 with phosphoric acid] (30:10:60, v/v/v); column: C18, 4.6 mm × 250 mm, 5 μm particle size; detection: UV at 227 nm

The figures in parenthesis are the corresponding theoretical values at *P* = 0.05

**Table 4:** One way ANOVA testing of the proposed methods and the USP official methods [4] for the determination of OLA and FLU in pure form:

	Source of variation	Sum of squares	Degree of freedom	Mean square	F	F critical
OLA	Between groups	0.07	1.00	0.07	0.06	4.96
	Within groups	13.37	10.00	1.34		
	Total	13.45	11.00			
FLU	Between groups	0.003	1.00	0.003	0.01	4.96
	Within groups	3.42	10.00	0.34		
	Total	3.42	11.00			

At 95% confidence level (*p* = 0.05), F < Fcritical indicates no statistically significant difference between the compared methods

**Table 5:** Comparison of analytical performance of proposed CM-SS/FZM-SS method versus reported spectrophotometric techniques:

	Reference (first author/year)	Analytical principle	Linearity range (µg/mL)	*R* ^2^	*R*%	%RSD	Remarks
1	Parmar et al. 2011 (Prajna)	Simultaneous Equation Method (Vierodt’s)	OLA: 1–12 FLU: 4–30	OLA: 0.9996 FLU: 0.9999	DF: OLA: 100.33-101.46 FLU: 98.64-103.21	Not validated acc. to ICH	Simple, but its sensitivity is generally poor due to high susceptibility to spectral interference, baseline noise, highly prone to error from minor wavelength shifts, all of which compromise accuracy and selectivity. making it unreliable for complex mixtures.
2	Parmar et al. 2011 (Prajna)	Absorbance Ratio (Q-Absorbance)	OLA: 2–16 FLU: 8–30	OLA: 0.9998 FLU: 0.9979 & 0.9999	DF: OLA: 97.24–98.64 FLU: 97.59–99.87	Not validated acc. to ICH	Resolution relies on a single isosbestic point, making the method sensitive to minor changes in pH or solvent effects, which can shift the point affecting the method’s robustness. Sensitivity is compromised due to reliance on the isosbestic point and the need for high wavelength accuracy.
3	Vekaria and Parmar, 2021	Q-Absorbance Ratio Method	OLA: 5–30 FLU: 10–60	OLA: 0.998 FLU: 0.999	OLA: 100.11-101.04 FLU: 100.37-100.38	OLA: Interday: 0.441 Intraday: 0.553 FLU: Interday: 0.325 Intraday: 0.556	
4	Parmar et al. 2011 (IJPSR)	First Derivative (D1)	OLA: 2–20 FLU: 8–80	OLA: 0.9993 FLU: 0.9991	OLA: 95.19–105.3 FLU: 98.32-100.09%	Not validated acc. to ICH	Offers better resolution than classic methods but the differentiation process amplifies noise, which severely compromises precision [potentially compromising precision and accuracy, especially at low concentrations.]
5	Tantawy et al. 2013	First Derivative (D1)	OLA: 5-17.5 FLU: 100–600	OLA: 0.9998 FLU: 1.00	OLA: 99.98 FLU: 100.33	OLA: Interday: 0.729 Intraday: 0.500 FLU: Interday: 0.270 Intraday: 0.252	
6	Tantawy et al. 2013	Derivative Ratio (DD1)	OLA: 5-17.5 FLU: 100–600	OLA: 0.9999 FLU: 0.9999	OLA: 100.04 FLU: 100.26	OLA: Interday: 0.743 Intraday: 0.721 FLU: Interday: 0.404 Intraday: 0.431	Highly effective resolution, but the multiple mathematical steps introduce complexity and potential cumulative error into the final result.
7	Ghanem et al. 2024	Dual wavelength ratio spectrum method	OLA: 4–20 FLU: 5–50	OLA: 1 FLU: 0.999	OLA: 100.72 FLU: 99.309	OLA: Interday: 1.149 Intraday: 1.229 FLU: Interday: 1.001 Intraday: 0.805	This method requires precise selection of four specific wavelengths and involves complex ratio subtraction, making it highly dependent on instrumental accuracy and susceptible to error propagation.
8	Proposed method	CM-SS / FZM-SS Spectrophotometry	OLA: 1.5–14 FLU: 3.5–35	OLA: 0.9999 FLU: 0.9999	OLA: 99.65 FLU: 100.27	OLA: Interday: 0.639 Intraday: 0.090 FLU: Interday: 0.756 Intraday: 0.249	- Fully validated according to ICH Q2(R1) guidelines demonstrating excellent precision and accuracy values. - Superior sensitivity reflected by narrow linearity ranges meaning the method can accurately quantify the drug at lower concentrations than the majority of published methods. - Statistical comparison to official methods showed no statistically significant difference. - Eliminating spectral interference without the noise penalty, combining the resolution of advanced methods with the stability of zero-order data, offering superior performance in the binary mixture.

**Table 6:** Robustness study of the proposed spectrophotometric methods using wavelength variation

Method	Wavelength variation*	Mean recovery (%)	SD
CM-SS	± 1 nm (at constant region 285–300 nm)	99.79	0.09
FZM-SS	282 ± 0.1 nm	98.99	1.86
FZM-SS	282 ± 0.1 nm	100.48	0.24

*For CM-SS, robustness was assessed by shifting the wavelength used to obtain the constant within the extended plateau region (285–300 nm). For FZM-SS, robustness was assessed by varying the selected zero-contribution wavelength (282 nm)

### Evaluation of the greenness of proposed methods using AGREE

**Fig. 6:**
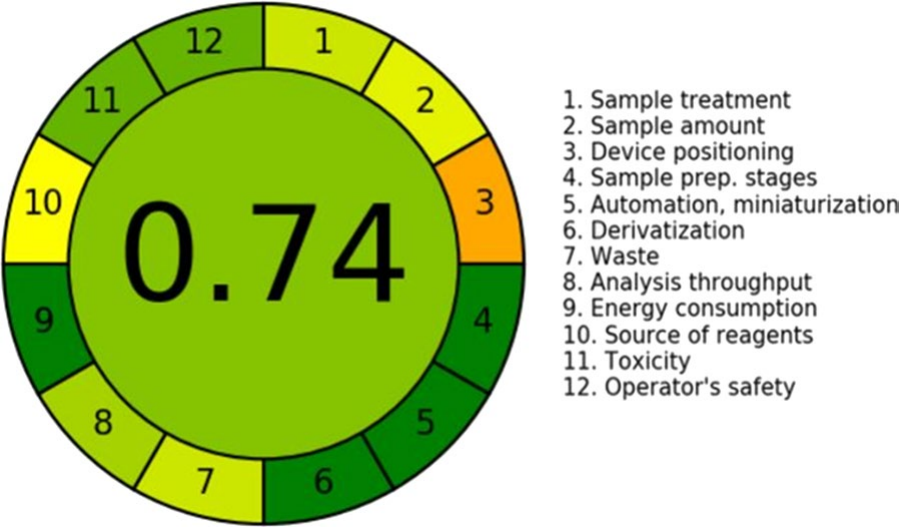
Greenness assessment of the proposed spectrophotometric methods according to the AGREE tool

Evaluation of the proposed methods’ greenness was achieved using the AGREE tool, which provided a comprehensive assessment of the proposed methods’ adherence to Green Analytical Chemistry (GAC) principles [48]. The AGREE tool, which is a downloadable software program, generates a visual representation in the form of a circular, color-coded pictogram. This pictogram is divided into twelve segments. Each segment corresponds to one of the twelve principles of green analytical chemistry [49]. The overall greenness score is displayed at the center of the circle, while the individual segments of the pictogram are shaded along a color gradient from deep green (indicating high compliance with green principles) to deep red (indicating low compliance with green principles), reflecting the environmental impact of each aspect. The proposed method achieved an AGREE score of 0.74, as illustrated in Fig. 6.

### Application of the RGB model for the WAC approach

The concept of White Analytical Chemistry (WAC) [50] is a newly introduced approach that focuses on assessing analytical methods based on efficiency in sample analysis, environmental sustainability, and practicality. This innovative approach is inspired by the color white, which results from combining the three primary colors: red, green, and blue (RGB). RGB models are used in the WAC approach to evaluate analytical methods. The red model focuses on evaluating the effectiveness of method validation based on four principles: linearity and sensitivity (R1), specificity (R2), accuracy and precision (R3), and robustness (R4). The green model takes into account the four fundamental GAC principles of measuring the greenness of analytical procedures, including waste generation (G1), energy consumption (G2), safety for humans and animals (G3), and the use of non-toxic, eco-friendly solvents (G4). The blue model assesses practicality and operational efficiency, including cost-effectiveness (B1), time efficiency (B2), the number of steps in sample analysis (B3), and the skill level required for instrument operation (B4).

While it incorporates GAC principles, WAC extends its evaluation beyond environmental aspects to also consider usability, affordability, and validity of the analytical techniques for drug analysis [51].

**Fig. 7:**
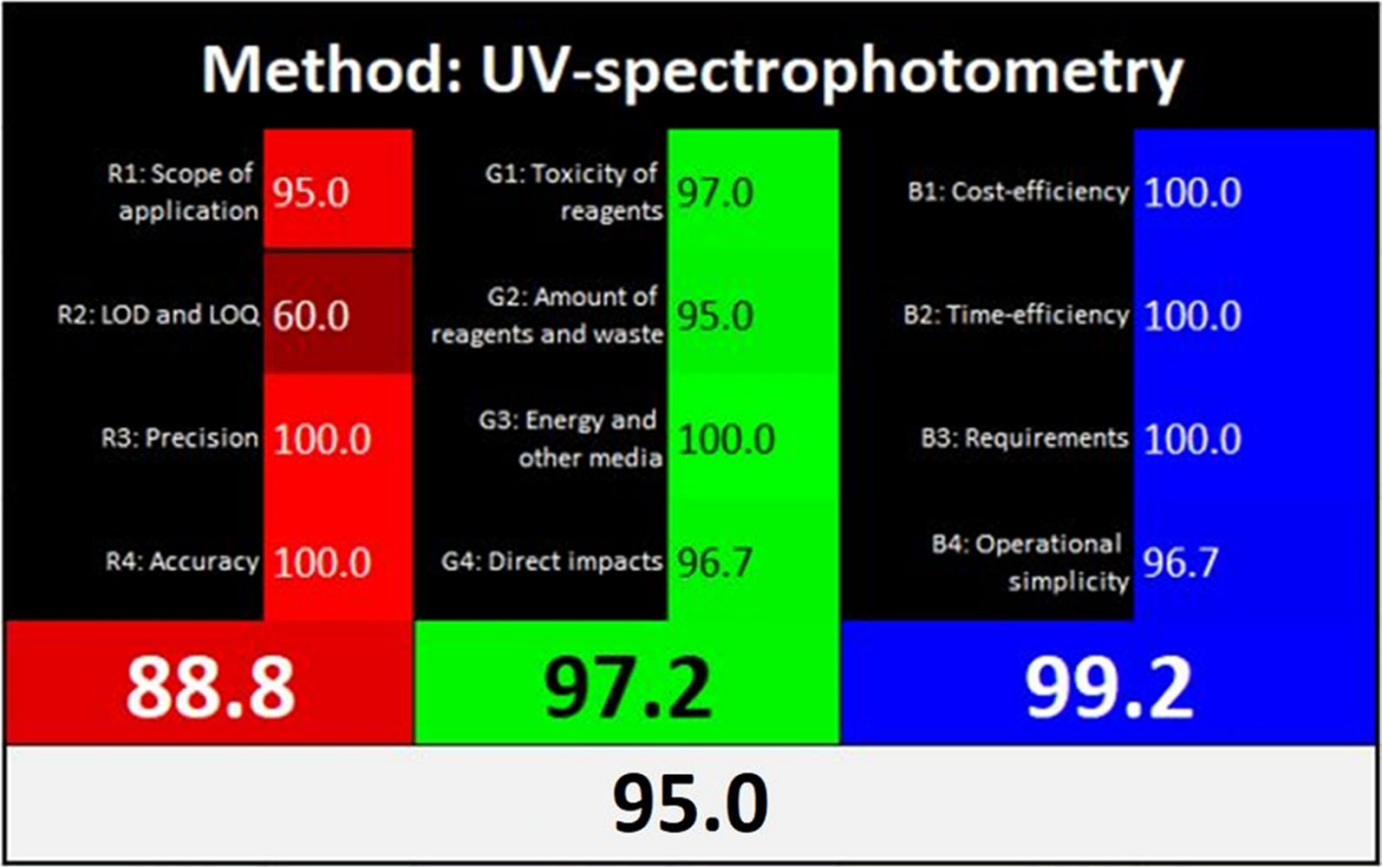
White Analytical Chemistry (WAC) approach for the assessment of validation efficiency, greenness profile, and practicality of the proposed spectrophotometric method using the red, green, and blue (RGB) paradigm

The validation efficiency of the proposed method was evaluated using the red model, with a score of 88.8, and this reflects a high degree of compliance with the ICH guidelines of validation. The green model was used to assess the greenness profile of the proposed method, resulting in a score of 97.2. The blue model was also applied to assess simplicity, time and cost-effectiveness. The proposed method achieved a score of 99.2; this is due to the use of low-cost, widely available reagents and simple, user-friendly instrumentation (UV-Vis spectrophotometer). Additionally, the use of a fast-scanning spectrophotometer contributed to the time efficiency of the method. The overall White Analytical Chemistry (WAC) assessment, integrating all three pillars (red, green, and blue), yielded an excellent white score of 95. The WAC approach-based evaluation of the proposed method is shown in Fig. 7.

The environmental benefits of the proposed methods were evaluated by comparing their greenness and whiteness metrics to those of previously published spectrophotometric methods for Olanzapine and Fluoxetine. A recent spectrophotometric study by Ghanem et al. (2024) [23], utilizing a dual-wavelength ratio spectrum method for the same drug combination, reported an AGREE score of 0.8 and an RGB WAC score of 88.9. Our proposed method achieved an AGREE score of 0.74 and a high White Analytical Chemistry (WAC) score of 95. The closeness of the AGREE score indicates that our method maintains a comparable level of environmental safety and sustainability to the currently reported methods. The choice of methanol: water (50:50, v/v) as the solvent system was a necessary requirement dictated by the fundamental solubility characteristics of the analytes. Both Olanzapine and Fluoxetine are practically insoluble in pure water [6–8]. Methanol was therefore essential to achieve molecular dispersion, ensure stability, and prevent precipitation. Compared to traditional chromatographic methods (such as HPLC or HPTLC), which often consume large volumes of highly toxic solvents like acetonitrile, ethyl acetate, or chloroform as the mobile phase, our method requires only a minimal, fixed volume of methanol for stock solution preparation. The overall solvent consumption per sample analysis is significantly lower, leading to remarkably reduced waste generation. While we explored the use of ethanol, a widely recognized greener alternative, its higher cost compared to methanol would significantly undermine the method’s core advantage of superior cost-effectiveness (WAC Blue Model, B1). Therefore, the methanol: water system was selected as the most practical and economically viable compromise to ensure both analytical performance and low operational cost.

Furthermore, the high WAC score of 95 strongly emphasizes the superior practicality, simplicity, and cost-effectiveness of our methods. This score is attributed to relying on simple zero-order spectra and minimal mathematical manipulation, effectively avoiding complex, multi-step derivative processes that compromise speed and precision. Moreover, the low operational cost is guaranteed by requiring only simple, widely available reagents, with no expensive chromatographic columns, and significantly lower consumption of organic solvents when compared to typical chromatographic mobile phases (e.g., acetonitrile/water mixtures). This comparison confirms that the CM-SS and FZM-SS methods are not only highly accurate but also represent an environmentally sustainable alternative for routine Quality Control (QC) analysis, offering clear, quantifiable advantages in terms of speed, simplicity, and low operational cost, establishing them as an excellent practical analytical choice.

## Conclusion

The proposed spectrophotometric methods were found to be rapid, cost-effective, and eco-friendly. They do not require complicated software or expensive or hazardous solvents. Although several spectrophotometric methods have been reported in literature for the simultaneous determination of OLA and FLU, the proposed methods offer superior performance by isolation and filtration of the spectra of each component to its D⁰ form, which effectively provides a distinct spectral profile for each drug.

SS has been shown to be highly effective when dealing with mixtures that display either partial or full spectral overlap, as it eliminates the entire spectrum of the interfering substance. Consequently, wavelength selection for calibration is not critical. SS was successfully combined with both CM and FZM. In the CM method, normalized spectra were used, so it needs an extended spectral region over which one of the components is contributing and the other is at zero absorbance. On the other hand, the FZM method relies on a single wavelength where one component shows contribution and the other exhibits no absorbance, making the process simpler.

The methods were shown to be accurate and precise at the λ_max_ of each analyte. They were also evaluated according to GAC and WAC principles, achieving high scores in terms of validation efficiency, environmental sustainability, and practical applicability.

Accordingly, the proposed methods could be successfully implemented as environmentally friendly, cost-effective analytical tools for routine analysis of OLA and FLU, whether in their pure form or in pharmaceutical formulations, particularly within quality control laboratories.

## Supplementary Information

Supplementary Material 1.

## Data Availability

The authors declare that the data supporting the findings of this study are available within the paper. Should any raw data files be needed in another format they are available from the corresponding author upon reasonable request.
