# Supplementary material for: Normalized vs factorized spectra: comparative spectrophotometric approaches for enhancing the resolution of zero-order absorption spectra of olanzapine and fluoxetine in mixtures
Source: BMC Chem. 2026 Feb 24;20(1):49. doi: 10.1186/s13065-026-01722-3 (PMC12954982; doi:10.1186/s13065-026-01722-3)
Supplement: Supplementary file 1 — Supplementary Material 1. [file 13065_2026_1722_MOESM1_ESM.docx]

| **Concentration**  **(µg/mL)** | **OLA** | | **FLU** | | **Constant**  **(285 – 300 nm)**  **“CM”** | **Amplitude at 282 nm**  **“FZM”** |
| --- | --- | --- | --- | --- | --- | --- |
|  | **CM**  **D^0^**  **226 nm** | **FZM**  **D^0^**  **226 nm** | **CM**  **D^0^**  **226 nm** | **FZM**  **D^0^**  **226 nm** |  |  |
| **Lab mixture**  **concentration**  **(FLU:OLA)** | **Recovery % ± SD** | | | |  |  |
| **20:7** | 100.33  ±0.22 | 100.96  ±0.22 | 99.24  ±0.20 | 98.80  ±0.20 | 7.12 | 0.38 |
| **25:6*** | 99.85  ±0.34 | 100.31  ±0.35 | 100.49  ±0.32 | 100.27  ±0.32 | 6.14 | 0.33 |
| **35:3** | 99.86  ±0.34 | 100.75  ±0.98 | 99.99  ±0.31 | 99.59  ±0.31 | 3.18 | 0.17 |
| **10:10** | 99.87  ±0.66 | 100.24  ±0.67 | 99.86  ±0.59 | 99.10  ±0.59 | 10.11 | 0.54 |
| **15:5** | 99.46  ±0.36 | 100.30  ±0.37 | 99.69  ±0.33 | 99.13  ±0.33 | 5.12 | 0.27 |
| **Dosage form**  **(Psycholanz®)**  **25:6** | **Recovery % ± SD** | | | | **Constant**  **(285 – 300 nm)**  **“CM”** | **Amplitude at 282 nm**  **“FZM”** |
|  | 99.80  ±0.33 | 100.26  ±0.33 | 100.44  ±0.31 | 100.22  ±0.31 | 6.10 | 0.32 |

**Supplementary Table S1:** **Detailed analytical parameters for CM-SS and FZM-SS methods for the determination of olanzapine (OLA) and fluoxetine (FLU) in laboratory-prepared mixtures and pharmaceutical formulation.**

Values of the constant obtained from the plateau region (285–300 nm) in the Constant Multiplication (CM) method and the amplitude at 282 nm in the Factorized Zero-Order Method (FZM) represent the mean values calculated from three replicate analyses (n = 3) for each laboratory-prepared mixture. Recovery results are expressed as mean ± SD of three determinations.
